# A case report of pulmonary hepatoid adenocarcinoma: promoting standardized diagnosis and treatment of the rare disease

**DOI:** 10.3389/fimmu.2023.1203876

**Published:** 2023-05-24

**Authors:** Kun Xu, Jin Gao, Lili Feng, Ying Fang, Xiuliang Tang

**Affiliations:** ^1^ Department of Medical Oncology, Jiangsu Cancer Hospital and Jiangsu Institute of Cancer Research and The Affiliated Cancer Hospital of Nanjing Medical University, Baiziting, Nanjing, China; ^2^ Department of Thoracic Surgery, Jiangsu Cancer Hospital and Jiangsu Institute of Cancer Research and The Affiliated Cancer Hospital of Nanjing Medical University, Baiziting, Nanjing, China; ^3^ Department of Ultrasonography, Jiangsu Cancer Hospital and Jiangsu Institute of Cancer Research and The Affiliated Cancer Hospital of Nanjing Medical University, Baiziting, Nanjing, China

**Keywords:** lung cancer, pulmonary hepatoid adenocarcinoma, diagnosis and differential diagnosis, immunohistochemistry, genetic testing

## Abstract

**Objective:**

To investigate the clinical features, pathological characteristics, immunophenotype, differential diagnosis and prognosis of pulmonary hepatoid adenocarcinoma using a clinical case and literature report.

**Methods:**

We analyzed the clinical presentation, histological pattern and immunohistochemistry of a case of primary hepatoid adenocarcinoma of the lung in April 2022. We also reviewed literature on hepatoid adenocarcinoma of the lung from PubMed database.

**Results:**

The patient was a 65-year-old male with smoking history, who was admitted to hospital with an enlarged axillary lymph node. The mass was round, hard, and grayish-white and grayish-yellow in color. Microscopically, it presented hepatocellular carcinoma-like and adenocarcinoma differentiation features, with abundant blood sinuses visible in the interstitium. Immunohistochemistry showed that the tumor cells were positive for hepatocyte markers, including AFP, TTF-1, CK7 and villin, and negative for CK5/6, CD56, GATA3, CEA and vimentin.

**Conclusion:**

Pulmonary hepatoid adenocarcinoma is a rare epithelial malignancy of primary origin in the lung with poor prognosis. Establishing the diagnosis relies mainly on the detection of hepatocellular structural morphology resembling hepatocellular carcinoma, and on clinicopathological and immunohistochemical testing to exclude diseases such as hepatocellular carcinoma. Combination treatment, mainly surgery, can prolong the survival of early-stage cases of the disease, whereas radiotherapy is mostly used for intermediate and advanced cases. Individualized treatment with molecular-targeted drugs and immunotherapy has shown different therapeutic effects for different patients. Further research is needed to better understand this rare clinical condition for the development and optimization of treatment strategies.

## 1 Introduction

Hepatoid adenocarcinoma (HAC) is a rare clinical condition that occurs outside the liver and has a similar morphology to that of hepatocellular carcinoma. It is highly malignant and aggressive, leading to a poor prognosis for patients. Pulmonary hepatoid adenocarcinoma (PHAC) is a rare epithelial malignancy of primary origin in the lung. The tumor can occur in many organs throughout the body, most frequently the stomach, but can also occur in the lungs. Establishing the diagnosis relies mainly on the detection of hepatocellular structural morphology resembling hepatocellular carcinoma, and on clinicopathological and immunohistochemical testing to exclude diseases such as hepatocellular carcinoma. Combination treatment, mainly surgery, can prolong the survival of early-stage cases of the disease, whereas radiotherapy is mostly used for intermediate and advanced cases. Individualized treatment with molecular-targeted drugs and immunotherapy has shown different therapeutic effects for different patients. Herein, we report a case of PHAC admitted to our hospital, analyze and summarize its clinical and pathological features, multi-omics data, immunophenotype, differential diagnosis, treatment and prognosis in the light of the literature for the first time, with the aim of improving clinical and pathologists’ understanding of PHAC for reference in clinical work.

## 2 Case presentation

### 2.1 General information

**Figure 1:**
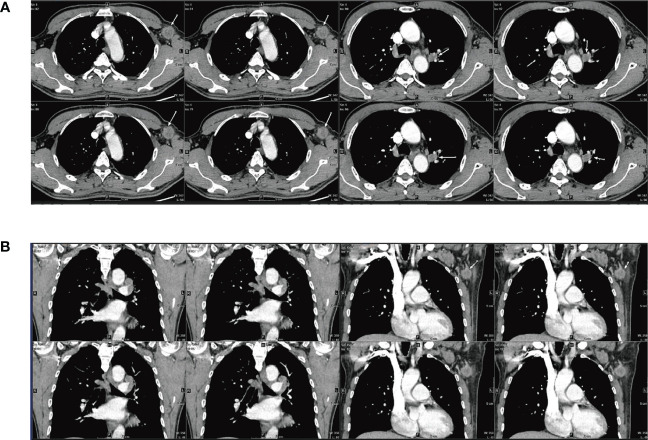
CT and MRI scan results for the patient **(A)** CT: Soft tissue nodular mass in the left upper lung adjacent to the mediastinum, measuring approximately 1.31×1.79 cm; multiple masses and enlarged lymph nodes in the aortopulmonary window, above the eminence, and both lung hilums; larger mass at the left lung hilum measuring 1.29×2.06 cm; (3) Mass with slight density visible in the left axilla, unevenly enhanced nodule is visible, measuring approximately 2.32×2.5 cm. **(B)** MRI: Dynamic contrast-enhanced MRI: multiple masses and enlarged lymph nodes in the aortopulmonary window, above the eminence, and both lung hilums; mass in the left lung hilum measuring approximately 1.6×1.6 cm; Enlarged lymph nodes visible in the left axilla, measuring approximately 2.4×1.9 cm.

**Figure 2:**
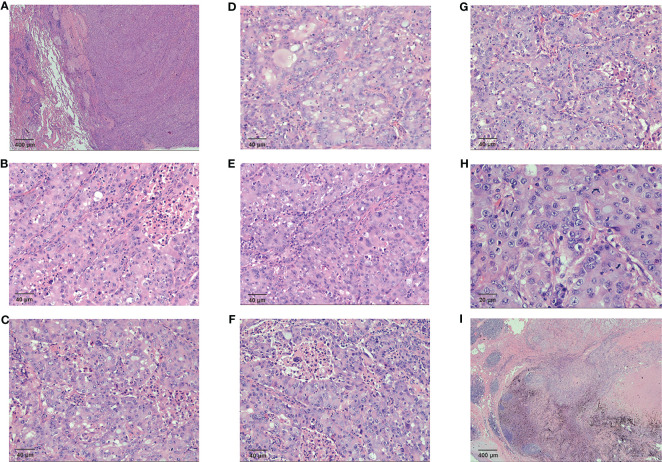
Pathological examination results for the patient **(A)** The fibrous border of the tumor (HE 20×) **(B)** Tumor cells were glandular in shape, with a nest-like adenoid structure (HE 200×) **(C)** Necrosis and phagocytosis were seen in the central part of the glandular lumen in some area (HE 200×) **(D)** Some cells were polygonal, with eosinophilic or clear cytoplasm (HE 200×) **(E)** Sieve-like structures were observed. Some of the cytoplasm and the central part of the glandular lumen had neutral mucus (HE 200×) **(F)** Abundant cytoplasm, biphasic, with distinct vesicular nuclei, small nucleoli, and apoptotic vesicles were visible (HE 200×) **(G)** Enlarged nucleoli and high nuclear grade were seen in some of the vesicular nuclei (HE 200×) **(H)** Some of the vesicular nuclei were enlarged and had high nuclear grade, with fine, open chromatin in the nuclei and pathological nuclear divisions (HE 400×) **(I)** The cancerous tissue directly invaded the hilar lymph nodes (HE 20×).

A 65-year-old male was admitted to our hospital in April 2022 for physical examination due to enlarged axillary lymph nodes and a mass in the left upper lung for a month. He had a history of smoking for more than 30 years, no history of hepatitis, and no other specific medical history such as gastric tumor. A chest CT and MRI showed a left upper lung mass indicative of a malignant lesion (**Figures 1A, B**). A lung puncture biopsy was performed, and metastatic adenocarcinoma was considered (**Figure 2**). Immunohistochemistry (IHC) of the biopsy suggested that it may have a biliary-pancreatic origin. PET-CT later revealed that: (1) soft tissue nodular hypermetabolic foci in the left upper lung adjacent to the mediastinum, with a high probability of malignancy; (2) multiple nodular hypermetabolic foci in the hilum bilaterally, with a high probability of mediastinal lymph node metastases; (3) nodular hypermetabolic foci in the left axilla. Serum AFP was 5820 ng/ml, and other tumor markers, including CEA, CA125, CA153, CA199 and NSE, were normal. Abdominal CT showed no abnormal masses in the liver, with possible multiple cysts in both kidneys and spleen. There were no abnormalities in the gallbladder, pancreas or abdominal cavity. No masses were palpable in the testes. Clinical diagnosis was left upper lung mass with a high probability of lung cancer.

### 2.2 Surgical treatment

The patient underwent thoracoscopic lobectomy with intrathoracic lymph node dissection, pulmonary artery trunk repair and radical axillary lymph node dissection in May 2022. 1.3 cm of the seventh intercostal space in the left axillary midline was used as the observation hole and 3.5 cm of the fourth intercostal space in the left anterior axillary line was used as the main operation hole. Intraoperative exploration revealed limited intrathoracic adhesions, no pleural effusion or abnormal nodules, and a 2.5 cm solid mass in the upper lobe of the left lung involving the pleura, with pleural folds. Enlarged lymph nodes of the first branch of the pulmonary artery were observed, surrounded by vessels, and enlarged lymph nodes of groups 5, 6, 7, 10, 11 and 12, were gray-black and hard. This was consistent with the preoperative diagnosis, and the decision was made to perform a left upper lung lobectomy.

The left inferior pulmonary ligament was dissected and released, the posterior mediastinal pleura in front of the pulmonary hilum was dissected, the left superior pulmonary vein was released and skeletonized, and the interlobular fissure was dissected. The left pulmonary artery trunk was dissected and skeletonized, the left superior pulmonary hyoid artery was further dissected and severed, and the interlobular fissure between the upper and lower lung lobes was closed and dissected using the endoscopic cutting suture device. The left superior pulmonary vein was closed and severed using the endoscopic cutting suture device. The left superior pulmonary artery was sutured with “4” surgical suture, and the anterior and posterior branches of the left superior pulmonary artery were closed and dissected using the endoscopic cutting suture device. The lymph nodes adjacent to the upper left pulmonary bronchus were cleared, closed and dissected with the endoscopic cutting suture device, and the bronchial stump was interrupted with 3-0 absorbable sutures.

The intraoperative rapid pathology report was “Left upper lung: carcinoma. Resection margin: no obvious malignancy”. Lymph nodes were further cleared. Postoperative pathological examination suggested a partial lobectomy specimen measuring 16.0 cm×9.0 cm×3.0 cm, with a round nodule measuring approximately 2.2 cm×1.7 cm×1.5 cm in size along the bronchial incision, with a grayish-white and grayish-yellow, hard surface and some areas of necrosis. Left upper lung: poorly differentiated carcinoma of the central type, pending IHC, no pleura was reached. Bronchial resection margin (-). Metastatic carcinoma was seen in the parabronchial (1/3) lymph node. Left axillary (1/1), group 11 (1/2), group 12 (1/1), 4L (1/1), metastatic carcinoma seen in the lymph nodes of group 11 (1/1), pending immunohistochemistry. No metastatic carcinoma was seen in lymph nodes of groups 5 (0/3), 7 (0/1), or 10 (0/1).

### 2.3 Specimen processing

Tissue specimens were fixed in 10% neutral formaldehyde, sampled according to standard procedure, dehydrated, paraffin-embedded, sectioned at 4 μm thickness, stained with conventional hematoxylin and eosin (HE) staining and IHC, and imaged by brightfield microscopy. The antibodies used for diagnosis and differential diagnosis were purchased from Beijing Zhong Shan-Golden Bridge Biological Technology Co., Ltd. IHC was performed using the EnVision two-step method and the staining step process was carried out strictly according to the kit instructions with positive and negative controls.

## 3 Results

### 3.1 Clinicopathological features

The HAC tissue had a fibrous border (**Figure 2A**). Tumor cells were glandular in shape, with a nest-like adenoid structure (**Figure 2B**). Necrosis and phagocytosis were identified in the centers of some glandular lumens (**Figure 2C**). Some cells were polygonal, with eosinophilic or clear cytoplasm (**Figure 2D**). Sieve-like structures characterized as neutral mucus in glandular lumens were observed, and intra-cellular features demonstrated abundant cytoplasm, biphasic, with distinct vesicular nuclei, small nucleoli, and apoptotic vesicles (**Figures 2E, F**). Sub-cellular pathology showed enlarged and highly atypical vesicular nuclei with fine, open chromatin and pathological nuclear divisions (**Figures 2G, H**). Moreover, the cancerous tissue directly invaded the hilar lymph nodes (**Figure 2I**).

### 3.2 IHC

**Figure 3:**
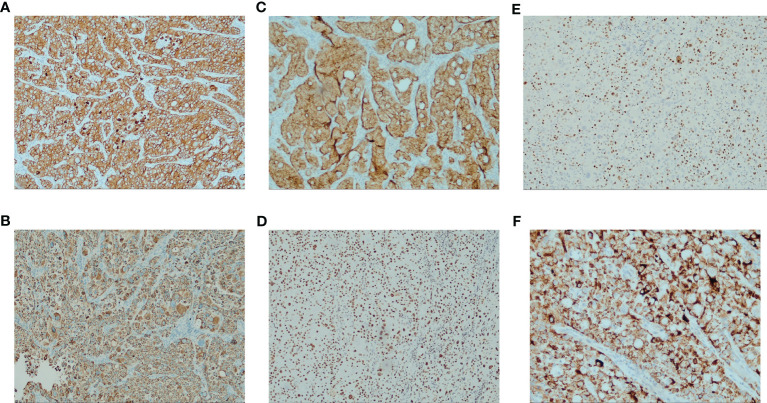
Immunohistochemistry results for the patient **(A)** CK7 strong positive (IHC [EnVision] 100×) **(B)** HepPar-1 positive (IHC [EnVision] 100×) **(C)** Villin positive (IHC [EnVision] 100×) **(D)** BRG1 (SMARCA4) Loss not detected (IHC [EnVision] 100×) **(E)** Ki-67 proliferation index > 50% (IHC [EnVision] 100×) **(F)** TTF1 characteristic features: intense cytoplasm staining and no nuclear stain (IHC [EnVision] 400×).

Left lung occupancy IHC marker results supported high-grade hepatoid adenocarcinoma. Pathological diagnosis was combined with clinical and other tests (SWI/SNF-deficient tumors were excluded). Results were: CK5/6-, p40-, CK7 3+, TTF1 cytoplasmic 2+, CD56-, S-100, Syn-, GATA3-, KUC43+, CD117 partial 2+, SALL4-, D2-40-, CD30-, D0G1-, Hep-12+, INI-1+, BRG1+, PD-L1(TPS)+50% <MXR003>, Ki-67 + 70%, ALK(V)-. Left axillary lymph node: GATA3-, villin2+, MUC42+, TTF1 cytoplasmic fraction 2+, SALL4-, CD117 foci+ (**Figures 3A–F**).

### 3.3 Genetic testing

Genetic testing covered a total of 111 genes, including the whole exome and specific intronic regions of eight commonly rearranged genes. Eight somatic mutations were detected, among which one had significant clinical significance, three had potential clinical significance, and four had unclear clinical significance. No pathogenic or likely pathogenic germline mutations were detected.

## Discussion

Pulmonary hepatoid adenocarcinoma is extremely rare, with only 60 published case reports included on Pubmed to date since its first presentation in the 1990s (**Table 1**). The tumor differs from common lung cancer subtypes in clinical presentation, pathological features and immunophenotype according to the latest WHO classification. Given the rarity and aggressiveness of the disease, a complete clinical presentation and morphological description, biological behavior and prognosis of any new case may contribute to the study and classification of the disease. Herein, we discuss pulmonary hepatoid adenocarcinoma in detail, in order to improve our understanding of this rare disease.

**Table 1:** Summary of publications on pulmonary hepatoid adenocarcinoma cases.

First Author, Year	Country	Sex	Age (Years)	Smoking (pack-year)	Location	Size(cm)	AFP	TNM	Treatment	Survival/ State
(1) Yasunami R, 1981	Japan	M	67	N/A	LUL	8	AFP↑	pT4N0M0 (IIIA)	R, I, C	16 months/dead
(2) Miyake M, 1986	Japan	M	55	N/A	RUL	5	AFP↑	pT4N2M1c (IVB)	RS	0.13 months/dead
(3) Tamura T, 1986	Japan	M	80	41	LLL	4.1 × 3.5	AFP↑	cT4N3M1a (IVA)	C	36 months/alive
(4) Miyake M, 1987	Japan	M	73	N/A	LUL	5 × 6 × 5	AFP↑	pT3N2M0 (IIIB)	RS, R	18 months/dead
(5) Saka H, 1988	Japan	M	73	N/A	RUL	3.9 × 3 × 3	AFP↑	pT2aN0M0 (IB)	RS	28 months/alive
(6) Kurimoto I, 1989	Japan	M	75	40	RUL	5	AFP↑	cT3N2M0 (IIIB)	N/A	N/A
(7) Okunaka T, 1992	Japan	M	49	Yes	RUL	6	AFP↑	eT3	RS	11 months/alive
(8) Yoshino I, 1996	Japan	M	54	N/A	RUL	2	AFP↑	pT1bN0M0 (IA2)	RS	24 months/alive
(9) Hirota F, 1999	Japan	M	80	45	RLL	5 × 4	AFP↑	pT2bN2M1b (IVA)	C	10 months/alive
(10) Carlinfante G, 2000	Italy	M	65	Yes	LLL	3.5	N/A	pT2aN0M0 (IB)	RS	84 months/alive
(11) Genova S, 2001	Plovdiv	M	71	N/A	LUL	7.7 × 6.4	N/A	pT4N0M0 (IIIA)	RS	24 months/alive
(12) Hayashi Y, 2002	Japan	M	55	87.5	RUL	5 × 4.8 × 6.5	N/A	pT3N0M0 (IIB)	RS	32 months/alive
(13) Terracciano LM, 2003	Switzerland	M	49	N/A	LLL	5	AFP↑	pT2bN0M0 (IIA)	RS	2 months/dead
(14) Oshiro Y, 2004	Japan	M	76	N/A	RLL	18 × 17 × 12	N/A	pT4N0M0 (IIIA)	RS	18 months/dead
(15) Ivan M, 2007	Canada	M	54	40	LUL + RUL	(13 × 11) + (3.3 × 2.6)	AFP↑	pT4N2bM1b (IVA)	C, R	N/A
(16) Kishimoto T, 2008	Japan	M	64	N/A	LLL	7.5 × 7 × 4	AFP↑	cT4N0M0 (IIIA)	RS	N/A
(17) Li CJ, 2008	China	M	65	N/A	RLL	6	AFP↑	cT4N2M1 (IVA)	TACE, TCM	16 months/dead
(18) Fornasa F, 2010	Italy	F	68	No	LUL	4.5 × 4 × 4	AFP→	pT2bN0M1 (IVA)	C	15 months/alive
(19) Kitada M, 2011	Japan	M	69	90	RLL	6.5	AFP↑	pT3N2M0 (IIIB)	RS, C	12 months/alive
(20) Mokrim M, 2012	Morocco	M	52	20	LUL	11.8 × 12 × 8	AFP↑	cT4N1M0 (IIIA)	C	7 months/alive
(21) Papatsimpas G, 2012	Greece	M	48	N/A	RUL	20 × 11 × 8	AFP↑	cT4N2bM0 (IIIB)	C, R	6 months/dead
(22) Valentino F, 2012	Italy	M	71	No	RLL	2.8 and 1.9	AFP↑	pT3N3M1b (IVA)	C, R, RS, bevacizumab	14 months/dead
(23) Cavalcante LB, 2013	Brazil	M	66	40	RLL	5 × 3	N/A	pT2bN0M0 (IIA)	Supportive treatment	0.4 months/dead
(24) Che YQ, 2014	China	M	66	70	LUL	5.3 × 4.6 and 7.9 × 10.0	AFP↑	pT4N0M0 (IIIA)	C, R	36 months/dead
(25) Haninger DM, 2014	America	M	51	45	RUL	4.2 × 3.7	N/A	cT2bN3M0 (IIIB)	No	14 months/dead
(25) Haninger DM, 2014	America	M	52	40	RUL	2.5	N/A	pT1cN0M1c (IVB)	No	37 months/alive
(25) Haninger DM, 2014	America	M	64	75	LUL	3.2 × 2.2	N/A	pT2aN0M1b (IVA)	No	10 months/dead
(25) Haninger DM, 2014	America	F	54	35	LUL	1	N/A	pT1aN0M1b (IVA)	C, R	108 months/alive
(25) Haninger DM, 2014	America	M	60	40	RUL	11.2 × 10.1 × 8.5	AFP↑	cT4N2M1b (IVA)	No	1 months/alive
(26) Shaib W, 2014	America	F	53	40	RUL	9.5 × 9.0 × 8.0	AFP↑	pT4N0M0 (IIIA)	RS, C	48 months/alive
(27) Al-Najjar H, 2015	England	M	71	30	RLL	Multiple	AFP↑	cT4N3M1a (IVA)	C	12 months/dead
(28) Gavrancic T, 2015	America	M	64	N/A	RUL	3.8 × 2.9	AFP↑	cT2aN2M1 (IVA)	C, Sorafenib, R	11 months/dead
(29) Grossman Kate, 2016	America	M	54	Yes	RUL	5.1 × 4.1	AFP→	cT3N0M1b (IVA)	C, R	3 months/dead
(30) Qian GQ, 2016	China	M	79	50	RUL	2.7 × 2.6	AFP↑	cT1cN0M0 (IA3)	Erlotinib	0.83 months/dead
(31) Sun JN, 2016	China	M	59	Yes	RUL	4.5 × 3.5 × 3.5	N/A	pT2bN0M0 (IIA)	RS	23 months/alive
(32) Wang S, 2016	China	M	56	N/A	RUL	4.0 × 4.1 × 4.8	N/A	cT4N1M0 (IIIA)	N/A	N
(33) Basse V, 2018	France	M	43	8	N/A	N/A	N/A	cTxN3M1c (IVB)	C, durvalumab anti- PD-L1 therapy	dead without following time
(34) Esa NYM, 2018	Malaysia	M	50	40	LUL	6 × 5 × 6	AFP↑	IIIB	R, C	7 months/dead
(35) Li Q, 2018	China	M	52	60	RUL	N/A	N/A	cT2N2M0 (IIIA)	C, R	2 months/dead
(36) Nakashima K, 2018	Japan	M	60	40	RUL	6.3 × 4.8	AFP↑	pT3N0M0 (IIB)	RS	8 months/alive
(37) Ruiz CD, 2018	N/A	F	69	70	LUL	8 × 8 × 5	AFP→	cT4N1M0 (IIIA)	R	1 months/dead
(38) Ayub A, 2019	America	M	61	40	RUL	2.3	N/A	pT1cN0M0 (IA3)	RS, R	6 months/dead
(39) Chen HF, 2019	China	M	53	No	RUL	5.3 × 3.5	AFP↑	pT3N0M0 (IIB)	RS, C, R, Icotinib, Osimertinib, Anlotinib	36 months/alive
(39) Chen Y, 2019	China	M	47	45	RLL	9.7 × 6.1 × 6.9	AFP→	cT4N3M1c (IVB)	C	2 months/dead
(40) EI Khoury A, 2019	England	M	59	>30	RUL	9.3 × 7.2 × 6.8	N/A	cT4N2M1b (IVA)	C	14 months/alive
(41) Kuan K, 2019	America	M	47	Yes	RUL	14	N/A	cT4N0M0 (IIIA)	RS	4 months/dead
(42) Li J, 2019	China	M	71	No	RLL	7 × 4.5	AFP↑	cT3N3M1b (IVA)	R	5.5 months/dead
(43) Malik SA, 2019	N/A	F	56	Yes	RLL	2 × 2	AFP→	N/A	N/A	2 months/dead
(44) Shi YF, 2019	China	M	60	No	RUL	7 × 7 × 5	AFP↑	pT3N2M0 (IIIB)	RS, C	15 months/dead
(45) Wang C, 2019	China	M	70	50	RUL	6.0 × 4.6	N/A	cT3N2M0 (IIIB)	C, R, Bevacizumab	9 months/dead
(46) Yang K, 2019	China	M	70	120	LLL	6 × 6 × 5.5	N/A	pT3N1M0 (IIIA)	RS	18 months/dead
(47) Chen JX, 2020	China	M	63	N/A	LLL+RUL	N/A	N/A	pT4N3M1c (IVB)	N/A	4 months/dead
(48) Chen LL, 2020	China	F	65	No	LL+RL	(7 × 5.1) + (9.2 × 4.6)	AFP↑	pT4NxM1b (IVA)	C, Bevacizumab, Anlotinib, Sintilimab	53 months/dead
(49) Muroyama Y, 2020	America	M	66	30	LUL	8 × 5	AFP↑	T4N3M1b	C+R	19 months/dead
(50) Tonyali O, 2020	Turkey	F	62	Yes	LUL	8 × 7 × 7 and 3 × 2.5 × 2	AFP↑	T4N1M0	RS +C+I+R	14 months/dead
(51) Chen Z, 2022	China	M	67	Yes	RML	7.1 × 5.3	AFP→	T4N1M0	C+ RS	13 months/dead
(52) Xu S, 2022	China	M	55	70	LUL	8.46 × 6.53	AFP↑	cT4N3M1a (IVA)	C+I	13 months/alive
(53) Yao Y, 2022	China	M	63	N/A	LUL	7.5 × 5.5	AFP↑	T4N0M0(IIIA)	RS	6 months/dead
(54) Galina G, 2022	America	M	54	25	RLL	Multiple (max = 14)	AFP↑	cT4N0M0 (IIIA)	C+I	7 months/alive
(55) Hou Z, 2021	China	M	66	Yes	RUL	3.3 × 2.5 × 4.0	AFP↑	cT2N2MO(IIIA)	C, R, Sorafenib +Sintilimab	13 months/dead
This report	China	M	65	Yes	LUL	16.0 × 9.0 × 3.0 (2.2 × 1.7 × 1.5)	AFP↑	cT1cN2aM0 (IIIA)	RS +C+Sintilimab	6 months/alive

*M, Male; F, Female; RS, radical surgery; C, chemotherapy; I, Immunotherapy; R, Radiotherapy; LL, left lobe; RL, right lobe; LUL, left upper lobe; RUL, right upper lobe; RLL, right lower lobe; LLL, left lower lobe; RML, Right middle lobe; AFP, Alpha Fetoprotein; N/A, Not applicable.

Hepatoid adenocarcinoma is a rare clinical condition with an aggressive extrahepatic tumor that morphologically resembles hepatocellular carcinoma. It was first named by Ishikura and colleagues in 1985 (56). In 2010, Metzgeroth and colleagues summarized the clinical features and case data of 261 cases of hepatoid adenocarcinoma, with the most frequent site of occurrence being the stomach (63%), with other cases being reported in the ovaries (10%), lungs (5%), gallbladder (4%), pancreas (4%) and uterus (4%) (57). There were also reports of hepatoid adenocarcinomas in the esophagus, duodenal papilla, jejunum, colon, rectum, peritoneum, thymus, mediastinum, kidney, renal pelvis, ureter and bladder. The prevalence is higher in males (male: female, 2.4:1), and the median age of onset is 65 years (21 to 88 years).

In 1990, Ishikura and colleagues studied seven cases of AFP-producing lung cancer and first proposed the name pulmonary hepatoid adenocarcinoma, five of which were confirmed to be hepatoid adenocarcinoma, and proposed diagnostic criteria for pulmonary hepatoid adenocarcinoma (58). The criteria were: (1) the presence of typical glandular or papillary adenocarcinoma; (2) the composition and expression of AFP are similar to those of hepatocellular carcinoma. However, in clinical practice, AFP expression was heterogenous in hepatoid adenocarcinoma cases. Previously, Haninger and colleagues proposed alternative diagnostic criteria for pulmonary hepatoid adenocarcinoma (25), including: (1) the tumor component can be purely hepatoid adenocarcinoma or hepatoid adenocarcinoma accompanied by typical glandular or papillary adenocarcinomas, imprinted cells, or neuroendocrine carcinoma; (2) positivity for AFP and other markers of hepatocellular differentiation is not essential; (3) adenocarcinoma with morphological features of hepatocellular carcinoma but without AFP production is referred to as AFP-negative hepatocellular lung adenocarcinoma.

Combining this case with the previous publications (**Table 1**), clinical symptoms of adenocarcinoma of the lung and liver are non-specific and similar to those of common subtypes of lung cancer. It usually starts with a cough and phlegm, chest tightness and shortness of breath, chest pain, and hemoptysis. Intrahepatic lesions are rarely identified, and patients have no history of liver disease, most patients have a smoking history, and the onset age is mostly over 50 years (38, 47). Most adenocarcinomas of the lung and liver are in a clinically progressive stage at the time of detection, and serum AFP is often elevated and correlates with disease activity. AFP levels often decrease after surgical removal of the primary tumors (58). For this patient with pulmonary hepatoid adenocarcinoma, preoperative AFP was 5820 ng/mL, which decreased to 1920 ng/mL five days after lobectomy AFP. Serum AFP was negative at 3 ng/mL on repeat chemotherapy in July, August and September, suggesting that AFP was produced from the tumor tissue.

Regarding pathological features, the tumors are usually large (up to 20 cm in diameter), grayish or gray-brown in color, well-defined, often necrotic, and could be enveloped by fibrous connective tissue. Microscopically, the tumors mainly consist of a hepatic differentiation region and a non-hepatic differentiation region. The tissue in the region of hepatic differentiation has nested masses and beam-like and sieve-like arrangements. Cancer cells appear to be large, polygonal, cytoplasm-rich, eosinophilic or hyaline, with large, PAS-positive nuclei. The interstitium inside the tumor tissue tends to be enriched in blood sinuses and often appears necrotic (20). Regions of non-hepatomatous differentiation are recognized as adenoid and papillary structures, with a few regions of poorly differentiated adenocarcinoma, some cases of steatosis or bile secretion, which may show imprinted cells or neuroendocrine differentiation (25).

Regarding immunohistochemical markers, AFP is a well-established marker for hepatocellular carcinoma, yolk sac tumor, embryonal carcinoma and other tumors of anterior intestinal origin. Elevated serum AFP or immunohistochemical expression of AFP is effective in the diagnosis of pulmonary hepatoid adenocarcinoma, but not all pulmonary hepatoid adenocarcinoma cases have significant AFP expression. Haninger and colleagues used a set of antibodies to compare the IHC features of lung metastases of hepatocellular carcinoma and five cases of pulmonary hepatoid adenocarcinoma. Co-expression of AFP, Hepar and CK8/18, and negative expression of CK14 were reported (25). In this HAC case, the positive expression of HepPar-1, AFP and CK7 is consistent with the expression of hepatocellular carcinoma. However, CK8, CK18 and CK19 were negative, which helps identify metastatic hepatocellular carcinoma. CK7 and CK19 are markers of bile duct epithelium, and in cases of intrahepatic cholangiocarcinoma, mixed hepatocellular carcinoma and/or hepatocellular carcinoma with bile duct differentiation, CK7 and CK19 are usually positively expressed at the same time. CK8 and CK18 are usually positively expressed at the same time in hepatocellular liver cancer. It suggests that immunohistochemical testing is of some value in identifying metastatic hepatocellular carcinoma. To sum up, our IHC staining showed distinguishing features of digestive system-originated adenocarcinoma, represented by positivity in HepPar-1, AFP, CK7 and MUC4. MUC4 is an immunogenic tumor-associated antigen (TAA) that elicits humoral and cellular immunity, especially in pancreatic cancer (59, 60). Some reports have suggested that MUC4 may be a promising candidate for immunotherapy (61). A peptide vaccine using MUC4 tandem-repeat glycopeptides-conjugated-tetanus toxoid induced an intense antigen-specific immune response in murine models (62). Research on herceptin reported that MUC4 prevented its specific binding to HER2 by steric hinderance (63, 64). Since both HER2 and EGFR are members of the ErbB family, this hepatoid adenocarcinoma case may not respond well to EGFR-targeted therapy. CD117 (c-kit) is expressed by hematological malignancies and mesenchymal neoplasms, and it was investigated as a druggable target for solid tumors as well (65). P40 and TTF1 are diagnostic markers for lung squamous cell carcinoma (LUSC) and lung adenocarcinoma (LUAD). Consistent with previous reports, this HAC case had TTF1 cytoplasmic fraction 2+ for both the primary lesion and axillary metastasis (25, 66). TTF1 expression in lung adenocarcinomas is normally localized within the nuclei, whereas TTF-1 cytoplasmic reactivity is found in hepatocellular carcinoma (67). The TTF1 cytoplasmic reactivity observed in the present and previous HAC cases suggests that TTF1 may play a role in maintaining the hepatic phenotype in HAC, and should be considered as a biomarker for HAC diagnosis (68). Ki-67 70% suggested active cell proliferation of tumor cells and good sensitivity to chemotherapy. Moreover, this tumor was positive for PD-L1 with a 50% tumor proportion score (TPS). Besides the above IHC characteristics, axillary metastasis was positive for villin, another indicator of adenocarcinoma of digestive tract origin.

There is currently no consensus on the origin of pulmonary hepatoid adenocarcinoma. The common view is that during embryonic development, the lung, liver and stomach are derivatives of the primitive foregut, and that certain tumors occurring in tissues and organs such as the lung and foregut may differentiate towards hepatocytes due to derangements in the differentiation process (69). Clinical findings confirm that tumors occurring in the above organs can produce certain products of normal hepatocytes or hepatocellular carcinoma, such as albumin, alpha-1 antitrypsin (AAT), lectins, ferritin, transferrin (TF), and AFP (70). In this case, the pathological pattern was diverse, with typical hepatic differentiation, visible glandular vesicular structures and heterogeneous differentiation. Presumably, the tumor stem cells had the potential for multidirectional differentiation, and the pulmonary hepatoid adenocarcinoma was the result of dysregulated differentiation of primitive multipotent stem cells into hepatocytes.

In clinicopathological practice, we believe that the following criteria, in addition to the diagnostic criteria proposed by Ishikura and Haninger, are also helpful in the diagnosis of pulmonary hepatoid adenocarcinoma: (1) elderly, male, smoker, large lung masses with varying degrees of elevated serum AFP; (2) areas of differentiation similar to that of the liver and/or areas of glandular vesicles or papillary structures are found in lung tumor tissue with a histomorphology similar to that of hepatocellular carcinoma; (3) positive IHC for AFP, hepatocyte, CK7 and/or CK8 and/or CK18 and/or CK19, TTF-1, etc.; (4) no previous history of liver disease and no imaging or clinical evidence of tumors in the liver or elsewhere.

At the same time, pulmonary hepatoid adenocarcinoma needs to be differentiated from the following tumors:

(1) Primary hepatocellular carcinoma with pulmonary metastasis: usually with a history of hepatitis and cirrhosis, and a mass can be found in the liver on imaging. Its morphology is mostly similar to that of pulmonary hepatoid adenocarcinoma. However, hepatocellular carcinoma does not show papillary structures in the histology, nor is it associated with neuroendocrine differentiation or intestinal differentiation. IHC shows expression of AFP and hepatocyte, but not CK5/6, CK20 and CEA (71).

(2) Common types of lung adenocarcinomas: most of the cells are glandular ducts or papillary structures, relatively uniform in size, round or oval, with abundant cytoplasm, often containing mucus, large nuclei, dark staining, often with nucleoli, and relatively clear nuclear membranes. CK7, CK8, CK18, CK19, TTF-1, Napsin-A, etc. can be detected through IHC (72).

(3) Squamous cell carcinoma of the lung: the morphological features are large, polygonal cells, more cytoplasm and darkly stained nuclei. In the more differentiated cases, the cells are arranged in multiple layers, and intercellular bridges and keratinized beads can be seen. In the moderately differentiated cases, the cells are large and polygonal, but there are no keratinized spheres or intercellular bridges. In the less differentiated cases, the cells are small, round or shuttle-shaped, and arranged in a non-hierarchical manner. Adenoid structures are not usually present. Positive expression of IHC CK17, P63, CK5/6, 34BE12, etc (73).

(4) Pulmonary metastasis of hepatoid adenocarcinoma from other sites such as the stomach: the morphology is similar, and the clinical presentation and general imaging are difficult to differentiate. In this case, no mass was seen in the stomach or other regions, and there was no history of relevant surgery, so pulmonary metastasis of hepatoid adenocarcinoma was not considered.

(5) Germ cell tumors: yolk sac tumors and embryonal carcinomas can show increased plasma AFP. There were no yolk cyst-like structures, and no testicular masses in this case. IHC was negative for CD30, ER and PR, so it could be excluded (74).

(6) Neuroendocrine carcinoma: the cells are composed of small to medium-sized cells with indistinct cytoplasmic boundaries, rounded and regular nuclei, arranged in sheets, cords, clusters, adenoid or chrysoidal clusters. IHC is positive for neuroendocrine markers including CD56, CD57, Syn, CgA, etc (75, 76).

**Figure 4:**
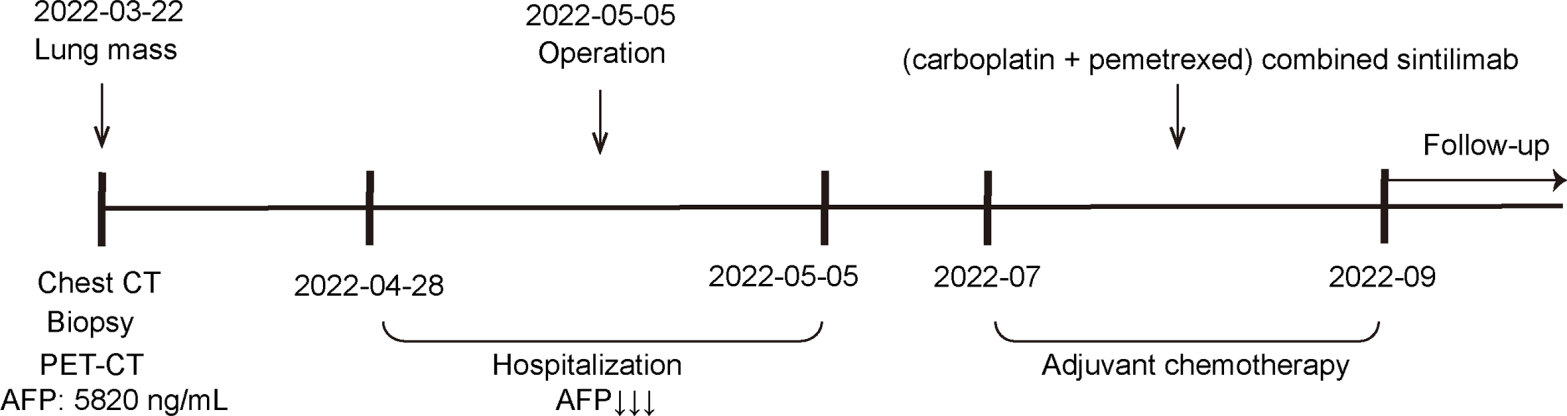
The clinical process comprising the patient’s treatment.

In terms of treatment, post-operative genetic testing was performed on the surgical specimen and peripheral blood. We identified co-occurring mutations in KRAS, STK11, CDKN2A, ATM, PTCH1, ARAF, SMO and MAP3K1, among which KRAS G12A is predictive of therapeutic sensitivity to MEK inhibitors and drug resistance to EGFR-TKIs (77). KRAS is an upstream signaling molecule of proliferative pathways such as the PI3K-AKT pathway and the RAF-MEK-MAPK pathway. It is a frequently mutated gene that occurs in 35% of lung adenocarcinomas with heterogeneous isoforms (78). Intriguingly, this hepatoid adenocarcinoma case corresponds with a previous large-scale survey on 3,560 patients that found that STK11 and ATM mutations are enriched in lung cancers with KRAS G12A mutation (77). CDKN2A mutation suggested the promising use of cell cycle inhibitors such as abemaciclib, palbociclib, and ribociclib. Additionally, other small molecules such as everolimus, which targets mTOR signaling pathway, and olaparib, which targets homogenous recombination deficiency, also suggested potential treatment effects based on genetic characteristics of this hepatoid adenocarcinoma case. Based on the results of the genetic test, we formulated a combination regimen of carboplatin 450 mg + pemetrexed 850 mg combined with sintilimab 200 mg on day 1, once every 3 weeks. After 3 months (four courses) of treatment, the efficacy was remarkable: the patient achieved stable disease after one month of treatment according to Response Evaluation Criteria in Solid Tumors v1.1. He was in overall good condition, had no recurrence and a negative serum AFP, and all tumor parameters were within normal range. The assessment on the follow-up visit in March 2023 showed that the patient had achieved a partial response, and he will continue to receive the planned treatment and supportive care (**Figure 4**).

Pulmonary hepatoid adenocarcinoma is clinically progressive and has a poor prognosis. It is prone to distant metastases to the liver, lung, bone and brain, as well as some uncommon sites such as the intestine, tonsils and gums (38, 47). Clinical staging of the disease is lacking, and no effective treatment has been established. A systematic analysis of pulmonary hepatoid cancer cases reported in the literature suggests that, as with other non-small cell lung cancers, the treatment of options for hepatoid adenocarcinoma of the lung remains surgery combined with chemotherapy and radiotherapy, when the conditions allow. Combination therapy, mainly surgery, can prolong survival in the early phases of the disease, and chemoradiotherapy is mostly used in the intermediate and late phases. Individualized treatment with molecular-targeted drugs and immunotherapy has shown different therapeutic effects in different patients and needs to be further explored. Of the cases with limited follow-up information reported in the literature (**Table 1**), 35 cases died between 4 days and 53 months after diagnosis, although a longer survival of 108 months was also reported (25). However, these data are still very limited, and further research is needed to elucidate the pathogenesis, diagnosis and comprehensive treatment to improve the prognosis of patients.

## Conclusion

This case report is the first multi-omic report that combines CT, PET-CT, MRI, pathological examination, IHC and genetic testing in the diagnosis and treatment of pulmonary hepatoid adenocarcinoma. We also discussed the diagnostic criteria and the differential diagnosis of pulmonary hepatoid adenocarcinoma based on the previously published cases, providing a reference for clinical practice. Our findings enriched knowledge about the clinical and pathological characteristics of this rare condition, and highlighted the importance of incorporating mutation status into treatment strategy, which raised critical points for consideration in further studies.

## Data availability statement

The original contributions presented in the study are included in the article/supplementary material. Further inquiries can be directed to the corresponding authors.

## Ethics statement

The patient provided informed consent to the publication of this article. Written informed consent was obtained from the individual for the publication of any potentially identifiable images or data included in this article.

## Author contributions

KX: Conceptualization, Writing — original draft, Investigation. JG, LF: Data curation, Validation. YF: Writing — review and editing. XT: Conceptualization, Supervision, Writing — review and editing, Investigation. All authors contributed to the article and approved the submitted version.
